# The sexual quality of life-female (SQOL-F) questionnaire: translation and psychometric properties of the Iranian version

**DOI:** 10.1186/1742-4755-10-25

**Published:** 2013-05-05

**Authors:** Raziyeh Maasoumi, Minoor Lamyian, Ali Montazeri, Seyed Ali Azin, Maria E Aguilar-Vafaie, Ebrahim Hajizadeh

**Affiliations:** 1 Department of Reproductive Health and Midwifery, Faculty of Medical Sciences, Tarbiat Modares University, Tehran, Iran; 2 Mental Health Research Group, Health Metrics Research Center,Iranian Institute for Health Sciences Research, ACECR, Tehran, Iran; 3 Sexual Transmitted Infections Research Group, Avicenna Research Institute, ACECR, Tehran, Iran; 4 Department of Psychology, Faculty of Human Sciences, Tarbiat Modares University, Tehran, Iran; 5 Department of Biostatistics, Faculty of Medical Sciences, Tarbiat Modares University, Tehran, Iran

## Abstract

**Background:**

Female sexual dysfunction is a common condition that extremely affects reproductive health and quality of life. To assess this health condition, a valid and reliable questionnaire is required. The aim of this study was to translate and validate the Sexual Quality of Life-Female (SQOL-F) questionnaire in Iran.

**Method:**

Forward-backward procedure was applied to translate the questionnaire from English into Persian. After linguistic validation and pilot examination, a cross-sectional study was carried out and psychometric properties of the Iranian version of questionnaire were tested. One hundred reproductive aged, married, healthy and sexually active women completed the questionnaire. Reliability was assessed by internal consistency (Cronbach’s alpha), and test-retest (intraclass correlation coefficient) analyses. In addition, content, and face validity were assessed and the factor structure of the questionnaire was extracted by performing exploratory factor analysis.

**Results:**

The mean age of participants was 33 (SD = 8.07) years, and the mean quality of sexual life score was 86.4 (SD = 1.78) ranging from 36 to 108. Most women were housewife (n = 92). Reliability evaluation revealed high internal consistency and good test-retest reliability. The Cronbach’s alpha coefficient was 0.73 and intraclass correlation coefficient (ICC) was 0.88. The mean scores for the content validity index (CVI) and the content validity ratio (CVR) were 0.91 and 0.84, respectively. The results of exploratory factor analysis (EFA) indicated a four-factor solution for the questionnaire that jointly accounted for 60.8% of variance observed.

**Conclusion:**

The findings from this study suggest that the Iranian version of SQOL-F questionnaire has good psychometric properties and it will be useful to assess the female sexual quality of life in reproductive health care settings.

## Background

Sex is a key function of human being and has a fundamental role in his or her reproductive life [1]. This function integrates physical, emotional and psychological factors and affects quality of life [2]. Indeed it has been suggested that any problems in sexual function might lead to a worsened general well-being and overall quality of life. However, there is limited, empirical evidence supporting this assumption [3]. Therefore measuring sexual quality of life is an important issue for assessing short- and log-term outcomes due to sexual problems [3-5].

The Sexual Quality of Life-Female (SQOL-F) questionnaire is a short instrument that specifically assesses the relationship between female sexual dysfunction and quality of life. Symonds and co-workers developed the questionnaire in 2005 [3]. The basis for the generation of the SQOL-F questionnaire was Spitzer’s Quality of Life (QOL) model that involved physical, emotional, psychological and social components. As such, the SQOL-F questionnaire was generated from semi-structured interviews studying a sample of 82 women, aged 19–65 years, from 7 countries: UK, US, Australia, France, Denmark, Holland and Italy.

Sexual problems are prevalent among Iranian women, as high as 26% to 51% [6-10]. Thus, attention to female sexual status and its effect on women’s quality of life are warranted in health care settings. To achieve this objective, a valid and reliable measure is required. Considering that at present there is no a valid Persian version (Iranian language) of the SQOL-F questionnaire, the objectives of this study were to translate and test the psychometric properties of the Iranian version of this questionnaire.

## Methods

### The SQOL-F questionnaire

The SQOL-F questionnaire is a specific and self-report instrument that focuses on sexual self-esteem, emotional and relationship issues. It consists of 18 items and each item is rated on a six-point response (completely agree to completely disagree). The response categories could be scored either 1 to 6 or 0 to5 giving a total score of 18–108 or 0–90. Higher score indicates better female sexual quality of life [3].

### Translation

After permission from the author (Symonds), the forward-backward procedure was applied to translate the SQOL-F questionnaire from English into Persian. For forward translation tow independent professional translators translated the questionnaire into Persian. Then, one of the author and translators compared Persian versions and produced a single Persian provisional version. In the backward stage, two other English experts translated the provisional Persian version back into English and provisional English version was produced. Afterward, an expert committee consisting of translators, one psychologist, two midwives and one epidemiologist compared the provisional English version with the original questionnaire and following linguistic and cultural adaptations, pre-final Persian version of the SQOL-F questionnaire was produced. This version was tested in a pilot study with 10 women and eventually, the final Persian version of the SQOL-F questionnaire was produced and it was used in this study.

### Design and data collection

This was a cross sectional study that carried out in Shiraz, Iran during April to July 2012. A sample of women attending to four maternal health and family planning centers affiliated to Shiraz University of Medical Sciences were entered into the study if they were at reproductive age (15–49 years), married, healthy, and sexually active. Sample size was estimated based on the number of items in the questionnaire multiplying by 5 (18 × 5 = 90) [11]. However, the actual sample size for this study was 100. After a short interview and explanation of the study, women who agreed to take part in the study completed the Iranian version of the SQOL-F questionnaire.

### Statistical analysis

Psychometric properties of the Iranian version of the SQOL-F questionnaire were assessed by several statistical tests as follows:

#### Reliability

The internal consistency of the questionnaire was assessed using the Cronbach’s alpha coefficient. Values equal or greater than 0.70 considered satisfactory [12]. In addition, test-retest reliability was conducted to assess the questionnaire stability estimating the intraclass correlation coefficient (ICC). Thirty participants completed the questionnaire twice in two-week intervals. ICC values of 0.40 or above were considered satisfactory (r ≥ 0.81-1.0 as excellent, 0.61- 0.80 very good, 0.41-0.60 good, 0.21-0.40 fair, and 0.0-0.20 poor) [11].

#### Validity

We assessed content, face and construct validity of the Iranian version of the SQOL-F questionnaire as follows:

1. Content validity: two methods (qualitative and quantitative) were applied. In the qualitative phase, an expert panel consisting of four psychometric experts and six health professionals including sexual and reproductive health specialists, health education experts, a maternal and neonatal health professional and an expert on social medicine assessed the content validity. Experts evaluated grammar, wording, item allocation and scaling of the questionnaire. In the quantitative phase, two indicators were calculated: the content validity index (CVI) and the content validity ratio (CVR). CVI assesses the relevancy, simplicity and clarity of an item to the content represented in an instrument [13,14]. For calculating the CVI a Likert-type, ordinal scale with four possible responses was used. The responses include a rating from 1 = not relevant, not simple and not clear to 4 = very relevant, very simple and very clear. CVI was calculated as the proportion of items that received a rating of 3 or 4 by the experts [15]. CVR examines the essentiality of an item in an instrument. For calculating this index the experts rate each item as essential, useful but not essential, or not essential [16].

2. Face validity: face validity was also studied by two methods: qualitative and quantitative. In the qualitative phase 10 women were asked to assess the questionnaire and indicate if they felt difficulty, or ambiguity in responding to the Iranian version of SQOL-F questionnaire. In the quantitative phase, we calculated the impact score (frequency × importance) to indicate the percentage of women who identified the item was important or quite important. Those items associated with an impact score equal or greater than 1.5 (which corresponds to a mean frequency of 50% and a mean importance of 3 on the 5-point Likert scale) were considered appropriate [17].

3. Construct validity: exploratory factor analysis (EFA) was performed to determine the underlying constructs of the questionnaire. As such principle components analysis (PCA) with varimax rotation was applied [18].

### Ethics

The Ethics Committee of Medical Sciences of Tarbiat Modares University, Tehran, Iran, approved the study. All participants were informed about the objectives of the study and were assured about confidentiality. Consequently, written informed consent was taken from all of them.

## Results

### The study sample

In all 128 women were approached and 100 agreed to complete the questionnaire. The main reason for refusal was the fact that 28 women did not like reporting their idea about sexual life. The mean age of participants was 33.0 (SD = 8.07) years. Most women were housewife (92%). The characteristics of study sample are shown in Table 1.

**Table 1:** Demographic characteristics of the study sample (n = 100)

		**Number**	**%**
**Age**	15-25	16	16
	25-35	42	42
	35-49	42	42
	Mean (SD)	33.0 (8.07)	
	Range	15-49	
**Educational status**			
	Primary	21	21
	Secondary	60	60
	College/University	19	19
**Occupational status**			
	Housewife	92	92
	Employed	8	8

### Sexual quality of life

The descriptive findings for the sexual quality of life scores are shown in Table 2. As shown women scored lower on the Sexual Repression (mean = 13.9, SD = 4.19) and Self-Worthlessness (mean = 15.4, SD = 3.72) subscales. The mean score for sexual quality of life was 86.4 (SD = 1.78).

**Table 2:** Descriptive statistic for the SQOL-F

	Mean score (SD)*	**Possible range**	**% from highest score**
**Psychosexual Feelings**	28.2 (7. 91)	7-42	67
**Sexual and Relationship Satisfaction**	24.3 (5.46)	5-30	81
**Self-Worthlessness**	15.4 (3.72)	3-18	85.5
**Sexual Repression**	13.9 (4.19)	3-18	77.2
**The SQOL-F**	86.4 (1.78)	18-108	80

* Higher values indicate better conditions.

### Exploratory factor analysis

Construct validity was evaluated by exploratory factor analysis (EFA). The Kaiser-Meyer-Olkin (KMO) and Bartlett’s test demonstrated that the data was appropriate for factor analysis (KMO index = 0.80, χ^2^ = 810.25, P < 0.001). Principal component analysis with varimax rotation identified four factors with eigenvalues greater than 1 and factor loading equal or greater than 0.4; accounting for 60.8% of variance observed. The factor loadings were as follows:

i. Factor 1 (Psychosexual Feelings) including 7 items (item 2, 3, 7, 8, 10, 16, and 17).

ii. Factor 2 (Sexual and Relationship Satisfaction) including 5 items (item 1, 5, 9, 13, and 18).

iii. Factor 3 (Self-Worthlessness) including 3 items (item 4, 6 and 15).

iv. Factor 4 (Sexual Repression) including 3 items (item 11, 12, and 14). The results are shown in Table 3.

**Table 3:** The results obtained from exploratory factory analysis of the Iranian version of SQOL-F

**Items**	**Factor 1**	**Factor 2**	**Factor 3**	**Factor 4**
2. ‘Frustrated’	**0.714**	0.483	0.235	0.096
3. ‘Depressed’	**0.700**	0.381	0.285	0.209
7. ‘Anxious’	**0.675**	0.101	0.467	0.197
8. ‘Angry’	**0.624**	0.011	0.275	0.247
10. ‘Worry’	**0.620**	0.017	0.315	0.235
16. ‘Worry of partner’s hurt or rejection’	**0.613**	0.051	0.266	0.325
17. ‘Feeling like losing of something’	**0.560**	0.122	0.334	0.328
1. ‘Enjoy’	0.078	**0.788**	0.133	0.187
5. ‘Good feeling about oneself’	0.120	**0.751**	0.171	0.216
9. ‘Closeness to partner’	0.189	**0.689**	0.156	0.096
13.’Talk to partner about sexual matters’	0.230	**0.648**	0.014	0.204
18. ‘Satisfaction with frequency of sexual activity’	0.003	**0.636**	0.107	0.491
4. ‘Feeling like less of a woman’	0.302	0.268	**0.754**	0.010
6. ‘Losing confidence’	0.316	0.232	**0.693**	0.259
15. ‘Feeling of guilt’	0.032	0.051	**0.633**	0.186
11. ‘Loss pleasure’	0.241	0.353	0.276	**0.746**
12. ‘Embarrassed’	0.231	0.086	0.121	**0.515**
14. ‘Avoiding’	0.203	0.132	0.423	**0.487**
*Explained Variance (%)*	*18.45*	*17.59*	*13.95*	*10.86*

Factor1: Psychosexual Feelings, Factor 2: Sexual and Relationship Satisfaction, Factor 3: Self-Worthlessness, Factor 4: Sexual Repression.

### Reliability

Reliability was evaluated using the internal consistency. The Cronbach’s alpha coefficient for the questionnaire was 0.73 and for its subscales ranged from 0.70 to 0.75; well above acceptable thresholds. In addition, the ICC was calculated and found to be 0.88 and for the sub-scales, ICC ranged from 0.50-0.88 (good to excellent), lending support to the stability of the questionnaire. The results are shown in Table 4.

**Table 4:** Reliability of the Iranian version of the SQOL-F

	**Number of items**	**Cronbach’ alpha**	**ICC**
**Psychosexual Feelings**	7	0.70	0.78
**Sexual and Relationship Satisfaction**	5	0.71	0.50
**Self-Worthlessness**	3	0.70	0.58
**Sexual Repression**	3	0.75	0.83
**The SQOL-F**	18	0.73	0.88

### Validity

The result of quantitative content validity showed that the CVI was 0.91 and the CVR was 0.84. In the qualitative phase four experts (25%) stated that a five-point response category is more suitable than the six-point rating scale. Other criteria such as grammar, wording, and item allocation were found to be appropriate. Quantitative face validity was examined by calculating the impact score. The index was equal or greater than 1.5 (ranging from 1.5 to 4.3) for all items. Therefore all items preserved for the following steps. In the qualitative face validity all participants acknowledged that they had no problems in reading and understanding the items.

## Discussion

As suggested ‘sexuality and reproductive capacity are fundamental aspects of being human. Our sexual and reproductive health and well being is as important to our quality of life as other key aspects of health such as physical and mental health and well being’ [19]. This study reported the psychometric properties of the Sexual Quality of Life-Female among a women population in Iran. Overall the results showed that the questionnaire is a valid and reliable instrument for evaluation of female sexual quality of life. The Cronbach’s alpha coefficient and intraclass correlation coefficient were acceptable and indicated good reliability and stability for the questionnaire. In addition, the CVI and the CVR indicated a reasonable content validity.

Validity of the SQOL-F questionnaire first was assessed in the UK and the USA. In the UK setting studying a sample of 1296 women aged 18–65 years, internal consistency was found to be 0.95 and the questionnaire discriminated well between depressed and not depressed women. Also, factor analysis showed that the questionnaire was a unidimensional construct because there was no obviously split of factor loadings [3]. In the USA setting studying three groups of women (women with spinal cord injury, women with sexual dysfunction and a sample of healthy women), the SQOL-F was lower among women with sexual dysfunction as expected lending support to its discriminate validity. In addition, intraclass correlation coefficient was reported to be 0.85, which showed an appropriate stability for the questionnaire [3]. A recent publication even confirmed that the SQOL-F showed a good convergent validity with the 28-item Sexual Function Questionnaire (SFQ28) [20].

The current study indicated a four-factor solution for the Iranian version of the SQOL-F, while the original questionnaire represents a unidimensional construct [3]. The four factors that we found were: 1. Psychosexual Feelings representing women’s feelings related to sexual experiences. 2. Sexual and Relationship Satisfaction indicating the concept of quantity and quality of sexual relationship and also, positive feelings about oneself and interpersonal relationship such as closeness. 3. Self-Worthlessness construct assessing negative feelings such as losing confidence and feeling of guilt. 4. Sexual Repression measuring loss of pleasure, embarrassment, and avoiding sexual activity. There might be several explanations for such different findings. For instance one might argue that different socio-cultural conditions might led women to respond differently to sexuality issues and thus difference on factor structure of the questionnaire in Iran and the UK in fact relates to women’s perspectives on sexuality and femininity. In Iran after a certain age most women should marry and thus sexual relationship outside of marriage is prohibited while in western culture a woman could remain single and thus having several sex partners might be allowed. In addition in countries such as Iran talking about sexual relationship or sexual problems by a woman might be seemed as rudeness while in western culture most women usually speak about these issues at ease. Another explanation for such observations might be related to labeling choice for the domains. For example the domains that built into the measure by the original developers are confidence, emotional impact and relationships and these are very similar in concept with worthlessness, psychosexual feelings, and relationship satisfaction, respectively. However, for determining definitive difference between constructs of the Iranian version and original scale of the SQOL-F, conducting the known-groups comparison, convergent validity and sensitivity analysis are suggested.

In general the sexual quality of life among the study sample was found to be acceptable. Yet, it seems that ‘Sexual Repression’ was low reflecting the need for further consideration in studying sexual quality of life among Iranian women. Finally, we think reporting overall sexual quality of life score instead of reporting each subscale would be better. Although scores for subscales could help to identify the area of concern in sexual relationship but since subscales are derived from the current study they could not be generalized. Similarly the instrument developers suggested that all inherent concepts of the instrument are heavily interrelated and should be assessed in an overall total score rather than as separate domains [3].

This study had some limitations. Firstly, the age range of women who participated is this study left within the reproductive period and pregnant and postmenopausal women were excluded. This restricted age period can be considered as one limitation. However, the reason for this sampling bias was due to the presence of a definitive difference among sexual status during reproductive ages, pregnancy and post menopause in Iranian women [21-24]. Secondly, a major limitation of our research is that we have not tested convergent or known-groups validity. However, the original validation study showed that the SQOL-F to have strong convergent and known-groups validity [3] and we would expect the same for the Persian version, especially given the solid results given above.

In conclusion the findings from this study indicated that the Iranian version of the SQOL-F questionnaire is a reliable and valid instrument for measuring female sexual quality of life.

## Competing interests

The authors declare that they have no competing interests.

## Authors’ contributions

RM collected the data, carried out the analysis and wrote the article. ML supervised the study and contributed to writing process. AM supervised the final translation, contributed to the study design and revised the manuscript. SAA contributed to the translation process and the study design. MV contributed to the translation process and reviewed the first draft. EH supervised the statistical analysis and contributed to the study design. All authors read and approved the manuscript.
